# Plastids: The Green Frontiers for Vaccine Production

**DOI:** 10.3389/fpls.2015.01005

**Published:** 2015-11-17

**Authors:** Mohammad T. Waheed, Hammad Ismail, Johanna Gottschamel, Bushra Mirza, Andreas G. Lössl

**Affiliations:** ^1^Department of Biochemistry, Faculty of Biological Sciences, Quaid-i-Azam UniversityIslamabad, Pakistan; ^2^NIBIO - Norwegian Institute of Bioeconomy ResearchÅs, Norway; ^3^Department of Applied Plant Sciences and Plant Biotechnology, University of Natural Resources and Applied Life SciencesTulln an der Donau, Austria

**Keywords:** molecular farming, biopharmaceuticals, plant-based vaccines, plastids, infectious diseases, developing countries, cost-effective vaccines

## Abstract

Infectious diseases pose an increasing risk to health, especially in developing countries. Vaccines are available to either cure or prevent many of these diseases. However, there are certain limitations related to these vaccines, mainly the costs, which make these vaccines mostly unaffordable for people in resource poor countries. These costs are mainly related to production and purification of the products manufactured from fermenter-based systems. Plastid biotechnology has become an attractive platform to produce biopharmaceuticals in large amounts and cost-effectively. This is mainly due to high copy number of plastids DNA in mature chloroplasts, a characteristic particularly important for vaccine production in large amounts. An additional advantage lies in the maternal inheritance of plastids in most plant species, which addresses the regulatory concerns related to transgenic plants. These and many other aspects of plastids will be discussed in the present review, especially those that particularly make these green biofactories an attractive platform for vaccine production. A summary of recent vaccine antigens against different human diseases expressed in plastids will also be presented.

## Introduction

Increase in the rate of infectious diseases is an escalating problem in both developed and developing world. There are number of factors that play role in the incidence rate of infectious diseases. Among these, main factors are global warming, lack of healthcare facilities, and costly preventive measures or treatments. With increase in global warming, threat of infectious diseases is also rising. Data from last 10 years show increase in the incidence of diseases and projections also predict more rise in future (Altizer et al., 2013). In developed countries, enhanced rate of infectious diseases may possibly be prevented by the availability of good healthcare facilities, cleaner resources and clean environment. However, in developing countries, where 2.2 billion people lived on less than US $2 a day in 2011 (World Bank, 2015), spread of infectious agents could be faster due to the reason that a large population is not able to afford costs related to treatments of these diseases. In addition, poor sanitation, malnutrition, use of unclean water and lack of precautionary measures at government levels are additional major contributors to the increase in the risks of infectious diseases.

Considering above mentioned factors, there are number of levels at which disease spread could be controlled. Two major levels include prevention and cure. Many diseases can be stopped more effectively by taking preventive measures. However, for eradicating existing infections and to control massive outbreaks of some infectious agents, cure is preferable. Keeping in view the scenario of developing countries, it is particularly important that these preventive/treatment strategies should be affordable and cost-effective. Use of vaccines can be an effective strategy that can be either used as prophylactic (before the onset of disease) or therapeutic (after the onset of disease). There are number of platforms that are used for antigen-based vaccine production, mainly mammalian cell culture based and fermentation-based systems. However, many concerns are related to the vaccines that are in market. Most important of these are cost, stability, safety, and efficacy. Hence, alternate strategies needs to be opted to cover the shortcomings of vaccines in use.

## Why chloroplasts?

There are various advantages of plant-based expression systems that are generally related to plants and some relevant to chloroplast-based expression only. In general, in relation to cost, a major advantage is at production level, i.e., the up scaling of plants to as much area as needed. If land is available, large number of plants can be grown by using inexpensive resources. This is the major level where cost is reduced in comparison to fermenters or bioreactors where establishing and running of a total new setup for up scaling is very costly. There are certain other advantages that are particularly related to chloroplast-based expression of vaccine antigens. Chloroplasts are organelles of plants present in leaves and other green parts that carry out photosynthesis. In recent biotechnological innovative era, chloroplasts have been explored for the expression of foreign proteins, industrially and pharmaceutically important compounds such as antibodies, growth factors, enzymes, hormones, cytokines, and antigens (Daniell et al., 2009; Lössl and Waheed, 2011). Previously, various aspects of plant/chloroplast-based pharmaceutical compounds have been extensively reviewed (Bock, 2014; Rybicki, 2014; Abiri et al., 2015; Chan and Daniell, 2015; Fahad et al., 2015; Salazar-González et al., 2015). Here we give an outline of chloroplast transformation and certain characteristics of plastids that are particularly important for the production of antigen-based vaccines against human diseases at low costs. We will review different aspects of chloroplasts for vaccine production particularly relevant to cost, stability, safety, and efficacy.

## Plastid transformation

There are mainly two methods that are used for plastid genome transformation: polyethylene glycol (PEG)-mediated transformation and gene gun-mediated transformation. First method is inexpensive and involves the isolation of protoplasts that are later transformed in the presence of PEG. However, the protoplast isolation is tricky and the protocols for protoplast isolation and regeneration are not optimized for many edible plant species. Transformation via particle delivery system (PDS) is expensive, both in the expenses of biolistic gun (although it is one time cost) and gold particles which are mostly used for the delivery of foreign DNA into plastomes. Despite, biolistic delivery is the most widely used method for chloroplast transformation and protocols are very well established (Verma et al., 2008; Abdel-Ghany et al., 2015). Different strategies have been adopted over time to enhance the foreign protein expression in chloroplasts attaining a very high amount of 72% of total leaf protein (TLP) from tobacco leaves (Ruhlman et al., 2010). These strategies include use of 5′ and 3′ untranslated regions or regulatory elements, use of active promoter, N-terminal fusion of a stabilizing peptide sequence and insertion site. Although there are very few reports showing very high expression of proteins in plastids, the expression levels in most studies of plastid transformation cross minimum level required for a feasible large scale commercial production, i.e., 1% of total soluble protein (TSP) or 50 μg/g fresh leaf tissue (Rybicki, 2009, 2010).

**Figure 1:**
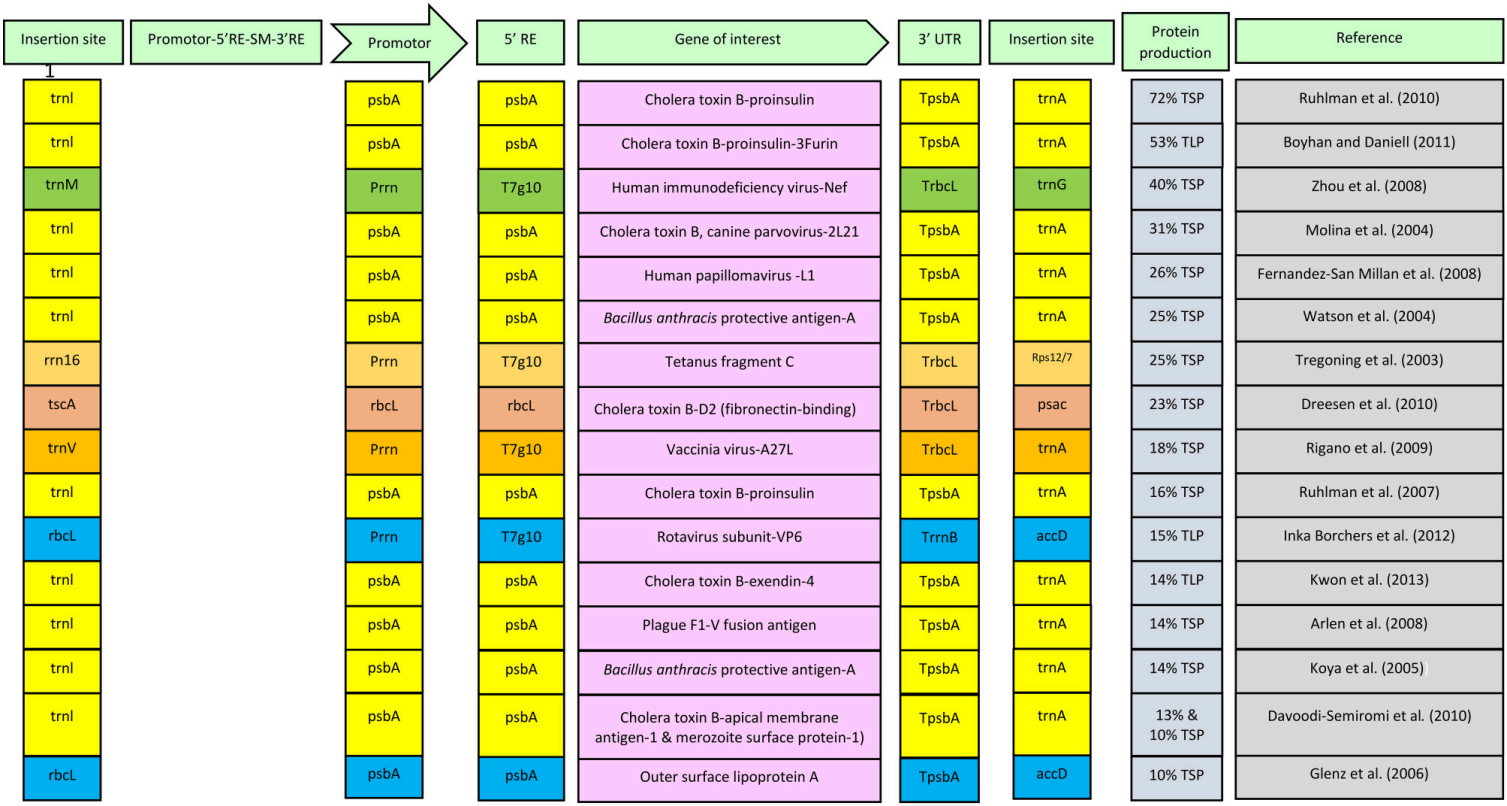
**Diagrammatic presentation of constituents of expression cassette along with their respective expression levels for vaccine antigens**. Combinations of similar insertion sites, promoters, regulatory elements and terminators are shown in one color. SM, selection marker; RE, regulatory elements; UTR, untranslated region; psbA, psbA gene; TpsbA, Terminator of psbA gene; rrn16, rrn 16 gene; T7g10, leader sequence of gene 10 of the lambda phage T7; rbcL, rbcL gene; TrbcL, Terminator of rbcL gene; TrrnB, *Escherichia coli* rrnB terminator; TSP, total soluble protein; TLP, total leaf protein.

Integration of expression cassette in plastid genome takes place via homologous recombination. Flanking sequences used for insertion on right and left sides are amplified from the species which is to be transformed and an expression cassette is constructed through various cloning steps (Verma et al., 2008; Bock, 2015). Choosing the insertion site for integration of expression cassette within plastomes is one of the important parameters for enhanced expression of transgenes. Different insertion sites can lead to different levels of expression. Two important parameters that should be kept in mind while choosing the insertion sites are their location in actively transcribed region and within the inverted repeat region of plastid genome (Verma et al., 2008). Insertion site can also have some negative effects on plants. Hence, to ensure safety of plant and enhanced expression of a vaccine antigen in plastomes, care should be taken in choosing the insertion site. Different insertion sites along with respective expression levels are shown in Figure 1. Since the flanking sequences are amplified from the plastome of the target species, a resulting species-specific vector is likely to express more in specific species for which it is designed. It is known that certain level of sequence homology exists between chloroplast genomes of different plant species (Bisaro and Siegel, 1980). On this basis, concept of universal vector was presented and it was proposed that a universal vector, having flanking sequences that are conserved in most plant species, can be constructed and utilized for transforming the plastomes of many related plant species (Verma and Daniell, 2007). However, using a universal vector may result in low expression. Hence to achieve high expression, a species-specific vector should be the choice. Nevertheless, it may be a technical challenge to develop species-specific vector for each particular species to be transformed because of lack of chloroplast genome sequence. A typical expression cassette of a transformation vector that is inserted in plastomes is shown in Figure 1. In this figure different components of expression cassette along with their respective yields of foreign proteins are also given. Data is given for those reports where the expression level is up to 10% or above of total soluble protein (TSP) or total leaf protein (TLP). Figure shows that highest expression for a vaccine antigen, which is 72% of TLP (Ruhlman et al., 2010), was achieved by using insertion sites trnI and trnA under the control of psbA promoter with 5′ regulatory elements from psbA gene. Most reports in the figure showing high expression of transgenes in plastids use this cassette containing trnI and trnA as insertion sites, promoter, 5′ regulatory elements and terminator from psbA gene. Hence it can be concluded that this expression cassette can serve as standard for attaining a high level expression in chloroplasts.

After transformation, several rounds of selection and regeneration are required on selection medium containing appropriate antibiotic to regenerate the homoplasmic transplastomic plants (Verma et al., 2008; Ahmad and Mukhtar, 2013). Homoplasmy refers to a state of plant when all chloroplasts are transformed and no wild type untransformed copy of plastid genome is left. However, to achieve homoplasmy, 1–2 extra regeneration cycles on selection medium are necessary. In comparison to mature leaves, very young leaves with immature chloroplasts have low plastome copy number. If these leaves are used for transformation, homoplasmy can be more quickly attained during regeneration phase. Normally, revised medium for organogenesis of plants (RMOP) is used, supplemented with appropriate concentration of hormones to promote callogenesis and shooting. For selection, antibiotic is added in the medium. Only those plants regenerate on selection medium which contain antibiotic resistant gene inside, i.e., transformed. Complete transplastomic plants are regenerated under aseptic controlled conditions and acclimatized to green house for further growth.

## Stable genetic resource

Once a complete transplastomic plant is regenerated via tissue culture, seeds are collected for growing next generation. These seeds can now serve as a stable genetic resource, an important outcome of stable genetic transformation. The costs involved in developing such resource are only one time, whether the developmental experimental procedures are carried out in academic research laboratories or at industrial scale. Currently, most of the research work regarding developing initial platforms and experimentations to develop techniques for enhancing the expression levels of foreign proteins in plants is carried out in academic research labs. Stable genetic resource in the form of seeds can be preserved and grown at any place where the vaccine production is required, exploiting a significant advantage of plants, i.e., “grown at site.” This aspect has potential to circumvent the costs related to transportations and cooling chain in those cases when vaccines are produced elsewhere and need to be transported to the target areas. Hence, it will ultimately reduce the cost of final product in the market.

## Polyploidy and very high expression of foreign proteins

An average tobacco leaf contains almost 100 chloroplasts per cell and in each chloroplast there are approximately 100 chloroplast genomes. In total, this makes 10,000 chloroplast genomes in one cell (Maliga, 2002; Koop et al., 2007). Theoretically, if all chloroplasts are transformed in each and every cell of every leaf in a plant, this makes very high expression of a foreign protein possible. In reality, a very high expression has been achieved, reaching up to 72% of total leaf protein (TSP) and 70% of total soluble protein (TSP), reported by Ruhlman et al. (2010) and Oey et al. (2009), respectively. For vaccine production at a cost-effective rate, this very high expression can play a key role. Higher the expression lesser will be the cost of final product because more product will be produced from less resources. The potential oral delivery of plant-based vaccines, which if possible in reality, will also greatly reduce the costs due to elimination of costly downstream processing. However, this does not seem to be a reality at the present stage due to number of limitations (for detailed discussion on this topic see Rybicki, 2009). In contrast, if the purification of protein has to be done then cost reduction is relevant only at production level, which is estimated to be about 31% (Rybicki, 2009). In such case, using the chloroplast-based expression and homoplasmy in which all plastomes are in transformed state, can lead to very high expression. This feature is expected to further reduce costs at production level because more protein will be produced per kilogram weight of plant. However, this also depends upon a number of other factors such as use of plants with wide leaves and having high biomass. In contrast to this characteristic of plastids, nuclear transformation, where mostly 1–2 copies of transgenes are mostly inserted in the nuclear genome, results in low yield (an average of 0.01–0.4% of TSP; Daniell et al., 2001a). Thus, for vaccine production, a high protein yielding platform such as chloroplasts needs to be opted. Various antigen-based vaccine candidates have been expressed in chloroplasts against number of human diseases. A detailed list has been previously published by our group (Lössl and Waheed, 2011). Reports published onwards are summarized in Table 1.

**Table 1:** **Different vaccine antigens against human diseases expressed via plastid genome since 2011**.

**Vaccine Antigen (Disease)**	**Expression system**	**Maximum expression level**	**Immunological investigation**	**References**
**VIRAL ANTIGENS**				
Dengue epitope region of E protein of DENV domain I and II (Dengue fever)	Lettuce	Not reported	Cross reaction of antibodies from the sera of dengue patients	Maldaner et al., 2013
Dengue-3 serotype capsid complete premembrane (prM) and truncated envelope (E) protein prM/E (Dengue fever)	Lettuce	Not reported	Not tested	Kanagaraj et al., 2011
Mutated human papillomavirus (HPV)-16 oncoprotein E7 (cervical cancer)	*Chlamydomonas reinhardtii*	Not reported	Vaccination in mice with algae extracts showed high level of E7-specific antibodies but low activation of E7-specific CD8+ cells	Vlasák et al., 2013
Mutated, attenuated E7 oncoprotein (E7GGG), alone or as a fusion with affinity tags (His6 or FLAG) (cervical cancer)	*Chlamydomonas reinhardtii*	0.12% of total soluble protein (TSP)	Induction of specific anti-E7 IgGs and E7-specific T-cell proliferation detected in C57BL/6 mice vaccinated with total Chlamydomonas extract and with affinity-purified protein	Demurtas et al., 2013
E7 translationally fused with β-glucuronidase	Tobacco	GUS-E7 showed expression between 30 and 40 times higher than previously reported for unfused E7 (0.1% of TSP)	Not tested	Morgenfeld et al., 2014
Modified HPV-16 L1 gene fused with glutathione-S-transferase (GST) GST-L1_2xCysM (cervical cancer)	Tobacco	Not detected	Not tested	Hassan et al., 2014
Synthetic gene encoding a C4V3 recombinant protein (HIV)	Tobacco	~25 μg/g of fresh weight	Plant-derived C4V3 has elicited both systemic and mucosal antibody responses in BALB/c mice, as well as CD4+ T cell proliferation responses	Rubio-Infante et al., 2012
Multiepitopic protein (Multi-HIV) carrying several neutralizing epitopes from both gp120 and gp41 (AIDS)	Tobacco	Protein accumulation levels up to 16 μg/g of fresh tobacco biomass	Multi-HIV protein was able to elicit humoral responses in mice when orally administered	Rosales-Mendoza et al., 2014
HIV-1 capsid protein p24 alone and in fusion with the negative regulatory protein Nef (p24-Nef) (AIDS)	Tobacco	P24 up to ~4% and p24-Nef up to ~40% of TSP	Subcutaneous immunization with purified chloroplast-derived p24 elicited a strong antigen-specific serum IgG response. Oral administration of a partially purified chloroplast-derived p24-Nef fusion protein, used as a booster after subcutaneous injection with either p24 or Nef, also elicited strong antigen-specific serum IgG responses	McCabe et al., 2008; Zhou et al., 2008; Gonzalez-Rabade et al., 2011
Rotavirus VP6 gene (gastroenteritis)	Tobacco	>15% of total leaf protein (TLP)	Not tested	Inka Borchers et al., 2012
**BACTERIAL ANTIGENS**				
Cholera toxin subunit B (CTB) fused with acid alpha glucosidase (GAA) CTB-GAA (Cholera, Pompe disease)	Tobacco	Between 0.13 and 0.21% of TLP	CTB-GAA fusion protein significantly suppressed immunoglobulin formation against GAA in Pompe mice	Su et al., 2015a
CTB fused with *Mycobacterium tuberculosis* antigens ESAT-6 and Mtb72F (a fusion polyprotein from two TB antigens, Mtb32 and Mtb39) (Cholera, TB)	Tobacco Lettuce	Maximum expression was 7.5% of TSP in mature tobacco leaves for CTB-ESAT-6	Hemolysis assay with purified CTB-ESAT6 protein showed partial hemolysis of red blood cells confirming the functionality of ESAT-6	Lakshmi et al., 2013
Major membrane protein I (mmpI) from *Mycobacterium leprae* fused with LTB (TB)	Tobacco	Not reported	Not tested	Hassan et al., 2013
EspA and Tir/Intimin antigens from enterohemorrhagic *E. coli* O157:H7 (hemorrhagic colitis)	Tobacco	Up to 1.4% of TSP	Upon oral administration of tobacco plant leaves high IgG and IgA specific antibodies were detected in serum and feces of mice	Karimi et al., 2013
Domain IV of *Bacillus anthracis* protective antigen gene [PA(dIV)] (Anthrax)	Tobacco	5.3% of TSP	Antibody titers of >10^4^ were induced upon intraperitoneal (ip) and oral immunizations with plant derived PA(dIV). Mice challenged with *B. anthracis* showed 60% and 40% protection upon ip and oral immunization with adjuvanted plant PA(dIV)	Gorantala et al., 2011
Anthrax protective antigen (PV) (Anthrax)	Tobacco	2.5–4% of TSP	Intraperitoneal and oral immunization with plant PA in murine model indicated high serum PA specific IgG and IgA antibody titers. Oral immunization experiments demonstrated generation of immunoprotective response in mice	Gorantala et al., 2014
**PROTOZOAN ANTIGENS**				
*Plasmodium falciparum* surface protein 25 (Pfs25) and 28 (Pfs28) (Malaria)	*Chlamydomonas reinhardtii*	0.5 and 0.2% of TSP, respectively	Antibodies to algae-produced Pfs25 were bond to the surface of *in vitro* cultured *P. falciparum* sexual stage parasites and exhibited transmission blocking activity	Gregory et al., 2012
*Plasmodium falciparum* surface protein (Pfs25) fused to the β subunit of the cholera toxin (CtxB) (Malaria)	*Chlamydomonas reinhardtii*	0.09% TSP	Algae produced CtxB-Pfs25 elicited CtxB-specific serum IgG antibodies and both CtxB- and Pfs25-specific secretory IgA antibodies	Gregory et al., 2013
*Toxoplasma gondii* surface antigen of (SAG1), alone and in fusion with heat shock Protein of *Leishmania infantum* (LiHsp83) SAG1, chLiHsp83-SAG1 (Toxoplasmosis)	Tobacco	0.1–0.2 μg/g fresh weight	Human seropositive samples reacted with chloroplast-derived SAG1, oral immunization in mice elicited significant reduction of the cyst burden	Albarracín et al., 2015
*T. gondii* GRA4 antigen (Toxoplasmosis)	Tobacco	0.2% of total protein	Oral immunization with chlGRA4 resulted in a decrease of 59% in the brain cyst load of mice compared to control mice. ChlGRA4 immunization elicited both mucosal immune responses	Yácono et al., 2012
Sexual stage antigenic surface protein Pfs48/45 antigen of *Plasmodium* (Malaria)	*Chlamydomonas reinhardtii*	Not reported	Not tested	Jones et al., 2013
**AUTOANTIGENS**				
Human proinsulin (A, B, C peptides) containing three furin cleavage sites fused with CTB (CTB-PFx3) (Diabetes type-1)	Tobacco Lettuce	47% of TLP in tobacco 53% of TLP in lettuce	Oral delivery of unprocessed proinsulin bioencapsulated in plant cells or injectable delivery into mice showed reduction in blood glucose levels similar to processed commercial insulin	Boyhan and Daniell, 2011
Human proinsulin gene fused with protein A (Diabetes type-1)	Tobacco	0.2% of TSP	Not tested	Yarbakht et al., 2015
Exendin-4 (EX4) fused with CTB (Diabetes type-2)	Tobacco	14.3% of TLP	Upon oral delivery in mice CTB-EX4 stimulated insulin secretion similar to the intraperitoneal injection of commercial EX4	Kwon et al., 2013

## Absence of epigenetic effects

Plastids are double membrane organelles that originated from prokaryotic symbionts and largely retained their characteristics of prokaryotes. Presence of double membrane enables the compartmentalized production of foreign proteins, thus retaining the proteins inside. Due to this reason, there are less chances of affecting the plant physiology by interfering with cellular metabolic pathways of plants. Related to their prokaryotic nature, an important fact is the absence of gene silencing and other epigenetic effects in plastids. Till date, there is no report of these effects taking place in plastids. This characteristic of the green organelles ensures the stable and continued expression of transgenes in chloroplasts. In contrast, nuclear genome is susceptible to epigenetic effects which affect the yield of foreign proteins.

## Expression of multigenes as single operon

Vaccines are often accompanied with adjuvants that boost the effect of a given antigen (Guy, 2007). Both chemical-based and biological adjuvants can be used for this purpose. *Escherichia coli* heat-labile enterotoxin subunit B (LTB) and cholera toxin subunit B (CTB) are two biological adjuvants that can be given with vaccine antigens to enhance their immunogenicity. Here, another advantage of plastids can play a role for production of vaccines coupled with biological adjuvants. Due to prokaryotic nature of plastids, multiple genes can be stacked as a single operon and co-expressed (Lössl and Waheed, 2011). Two or more genetic sequences can be stacked one by one and expressed under a single promoter. Utilizing this characteristic, coupled expression of a biological adjuvant with an antigen in chloroplasts can be achieved. In this way, costs related to separate production of adjuvants can be eliminated. In addition, direct coupling is believed to enhance the immunogenicity of antigens more than their separate administration (Guy, 2007; Sánchez and Holmgren, 2008) and it has been found that due to coupling of antigen with CTB, much strong response is achieved upon oral administration (Guo et al., 2012). This characteristic of plastids to express many coding sequences as single operon can also be utilized to develop bivalent to multivalent vaccines, in which two or more vaccine antigens against different diseases can be co-expressed. Development of multivalent vaccines, used to cure multiple infections with one vaccine formulation can be particularly important in case of patients with acquired immunodeficiency syndrome (AIDS) where multiple diseases may need to be cured at one time. This advantage of plastids is also applicable for those vaccines that need more than one epitope for their function.

Another advantage of using biological adjuvants and their direct coupling is potential safety and efficacy. Use of biological adjuvants will help to eliminate toxic chemical adjuvants such as aluminum hydroxide and aluminum phosphate which commonly cause many adverse side effects such as local irritation and carcinogenesis (Gupta and Siber, 1995; Sun et al., 2006). Thus, use of biological adjuvants addresses the safety concern. Additionally, direct coupling of adjuvants is advantageous in terms of efficacy of antigen. Direct coupling not only can enhance the immunogenicity of an antigen but also the adjuvanticity is presumed to be more pronounced in an adjuvant-antigen couple. Furthermore, if an adjuvant such as LTB is directly linked with an antigen, it can facilitate the entry of antigen through gut mucosa, bind to GM1-ganglioside receptors and aid in eliciting the protective immunity (Granell et al., 2010; Salyaev et al., 2010).

## Safety of plants/chloroplast-derived vaccines

Safety concerns arise at two levels related to plant-based vaccines: the environment safety and safety of final product for patients. To successfully launch these vaccines into market, both these concerns are essential to address in order to get the approval from competent authorities.

## Maternal inheritance of plastid genome

A major concern related to regulatory approval of plant-based pharmaceuticals is environment safety. Environment can be potentially contaminated by unwanted flow of transgenes, especially antibiotic resistant marker genes, to the wild species via pollen or to the environment or food chain vertically as well as horizontally. Certain aspects of environment safety concerns can be addressed by opting plastid transformation. In most plant species, plastids follow maternal inheritance pattern, i.e., not transferred through pollen. This is a major advantage of plastids that should aid in addressing the regulatory concerns related to genetically modified plants (GMPs) because there will be no or very negligible outflow of plastid DNA paternally (Daniell, 2007; Ruf et al., 2007; Svab and Maliga, 2007). In case of tobacco, there is an extra advantage that it is a non-food non-feed crop that is not very common as wild. Hence, chances of crossing transplastomic plants with any wild species are excluded. Generally, the risk of horizontal gene transfer from plants to microorganisms, particularly when transformed plants contain antibiotic resistance genes, is very low and there is no existing report of such incidence (Obembe et al., 2011). The risk goes more toward downside by the fact that plants naturally harbor many bacteria that contain antibiotic-resistant genes (Nielsen et al., 1998). Hence, it can be argued that horizontal gene transfer to soil bacteria is very negligible. Considering the advantage of chloroplast transformation related to transgene containment, it is very likely that field trials can be allowed in isolated areas at local level in developing countries after addressing the containment issues to the local authorities. Permission of field trials of transplastomic plants may be easier in case of non-food and/or non-feed crops such as tobacco, because in such case the risk of human food chain contamination is minimal. If safety concerns are addressed, field trials are allowed and have been done previously in various countries for plant/chloroplast derived recombinant proteins and biopharmaceuticals (Arlen et al., 2007; Hefferon, 2015).

Plant-based vaccines have the potential to be used for oral administration. However, a matter of concern is that whether the consumption of plant-made pharmaceuticals is safe for administration or not. In general, plants are safe for human consumption as plants are not host for human pathogens and many plant species such as lettuce are consumed in normal diet as raw. However, for consuming transgenic plants, safety needs to be addressed. There are several reports in which safety of plant-based pharmaceuticals have been shown in animal models via oral delivery against different diseases using raw plant material (for review see Lössl and Waheed, 2011; Chan and Daniell, 2015). However, in addition to product used for treatment, raw material also contains unwanted antibiotic resistant gene that raises concerns regarding safety and needs to be addressed in humans. This problem can be covered by: using selection markers other than antibiotic resistant genes, excising the antibiotic resistant genes after selection (Iamtham and Day, 2000) or purifying the final product. Although purification will add to the cost, yet it will address an important regulatory concern. In context of purification of plant-based vaccines, it is pertinent to mention that these vaccines will require less stringent purification methods compared to fermenter-based systems.

## Stability of plant/chloroplast-expressed proteins

In developing countries, a major limitation can be the maintenance of cooling chain during storage or when delivering the vaccines to remote areas. This factor may additionally add to the costs related to the production of vaccines. Plant-based vaccines have a potential that these can be stored at room temperature in the form of dried material for longer period of time. There are many reports which show that the protein expressed in plants remained stable at room temperature or even at elevated temperatures for a longer period of time when stored as dried plant material. An algal chloroplast-derived vaccine antigen remained stable for 20 months at room temperature in lyophilized form and was comparably immunogenic when tested in comparison with the antigen stored at 4°C (Dreesen et al., 2010). In another report, transplastomic lettuce leaves expressing CTB fused with ESAT-6 (antigen from *Mycobacterium tuberculosis*) were lyophilized and stored for 6 months at room temperature (Lakshmi et al., 2013). After 6 months CTB-ESAT6 fusion protein was stable and preserved proper folding, disulfide bonds and assembly into pentamers. Lyophilization also increased the antigen concentration per gram of leaf tissue up to 22-fold. Gregory et al. (2013) demonstrated that CtxB-Pfs25 accumulated as a soluble, properly folded and functional protein within algal chloroplasts, and it was stable in freeze-dried alga cells at ambient temperatures. Kwon et al. (2013) expressed exendin-4 (EX4) as a cholera toxin B subunit (CTB) fusion protein. They observed that lyophilization of leaf material increased therapeutic protein concentration by 12- to 24-fold, extended their shelf life up to 15 months when stored at room temperature and eliminated microbes present in fresh leaves. In addition, the pentameric structure, disulphide bonds, and functionality of CTB-EX4 were well preserved in lyophilized materials (Kwon et al., 2013). In a recent report Su et al. (2015a) showed that plastid-derived cholera toxin subunit B fused with acid alpha glucosidase (CTB-GAA) concentration was increased by lyophilization to 30-fold (up to 190 μg per g of freeze-dried leaf material). Same group (Su et al., 2015b) expressed coagulation factor IX (FIX) fused with CTB in commercial lettuce. They showed that CTB-FIX in lyophilized cells was stable with proper folding, disulfide bonds, and pentamer assembly when stored for approximately 2 years at ambient temperature. All these reports strengthen the fact that plant/chloroplast-produced biopharmaceuticals can be stored at room temperature for long periods of time in the form of lyophilized material. A vaccine may be very effective in the laboratory; however, its commercial potential can be limited unless suspension can be stabilized for storage and distribution. Alternative techniques such as establishment of a cold chain may cause the potential loss of vaccine stocks resulting from freezer failure and also adds to the costs when distributing frozen materials. Lyophilization (freeze-drying) is a well-established technique used in the pharmaceutical industry for stabilizing high-cost, labile bioproducts, such as vaccines (Adams, 2003). Firstly, plant-produced vaccines can be stored in the form of dried plant material. This will also increase the antigen concentration. Alternatively, plant-derived vaccines can be purified and lyophilized for storage at room temperatures and transported when required. This strategy will help to circumvent cooling chain in developing countries and thus eliminating the costs related to storage and transportation under cooling.

## What next?

It has been almost 14 years since the expression of first vaccine candidate antigen against human diseases in chloroplasts of *Nicotiana tabacum* (Daniell et al., 2001b). Since then lot of research has been done on different aspects of expression that has resulted in improved protocols and a very high expression level has been achieved in chloroplasts. In 80–90% of cases, tobacco is used for the chloroplast-based expression and comparatively very little research has been done on other plant species. For tobacco, excellent data is available to follow for chloroplast-based vaccine production. However, despite of all this research, not a single plant/chloroplast-based vaccine against human diseases has entered the market and hence humanity has not been benefited. What is needed but lacking is more industrial interest. Big pharma companies have already well-established platforms for the production of pharmaceutical compounds. A good amount of revenue is already generated from these systems. To adopt plant-based systems, pharmaceutical companies would need to invest money and resources to develop their own platforms. In addition, clinical trials and the ultimate regulatory approval would also require cost and time. In contrast, newly established pharma companies or small industries can adapt more quickly to plant-based systems because opting to new cost-effective systems will help these industries to compete and earn more compared to the expenditures. Another possible solution can be to establish and strengthen the research/academia-industry linkages. Research groups in collaboration with industry can develop platforms that could be later taken over by the industries. Such collaborative projects can be funded by many organizations that are working for the control of diseases throughout the world. Since, many cost-effective vaccines are needed mainly in developing countries, initially platforms can be established directly in those countries where vaccines are mostly needed. Small pharmaceutical industries can be collaborated to run the projects directly in a developing country where the product is to be utilized. Growing of plants, harvesting, processing and packaging can be carried out locally that will be economical due to less labor and land costs as compared to developed countries. A possible attraction for the pharmaceutical companies can be an adequate amount of revenue that can be generated from the production of vaccines and its launch to previously uncovered areas. This will be especially valid in those cases where already marketed vaccines such as those against HPV are very expensive and hence the large market of developing world remains uncovered. An affordable alternative solution in such cases can bring a handsome amount of turnover to the companies. The involvement of local pharma will be necessary because production can be more cost-effective if manufactured locally rather than exporting from a distant industrially developed country. In this way costs related to transportation and maintenance of cooling chain will be circumvented. Another advantage of this strategy will be the strengthening of local small pharmaceutical industries that will also help in the economic uplift of low and middle income countries. To achieve this, technology can be established in the developed countries in the laboratories/industries. Thus, a stable genetic resource in the form of seeds can be maintained in developed countries where more funding is available for research. The technology can be initially patented where developed. Later, local pharmaceutical companies, local governments and/or humanitarian organizations can be engaged to set up the industrial scale production platforms. Involvement of local governments and organizations such as world health organization (WHO) can serve to utilize already existing setups of vaccine storage, transportation and administration. However, a local patent may be needed if license is already not obtained in developing country, for which some variations in the existing established protocols of expression may be needed. To accomplish these goals, already established tobacco prototypes may be taken and advanced to the industrial level. Two main advantages of adopting tobacco chloroplast transformation: non-edible nature of tobacco thus avoiding food chain contamination and lack of transgene transmission due to maternal inheritance of chloroplasts may facilitate to address biosafety concerns in the target developing country and thus making large scale production in the fields possible. Established protocols, high level of expression and high biomass are additional reasons that make this species ideal for vaccine production at large scale. Other possible alternative plant species may be lettuce due to its high biomass and broad leaf. Taking lettuce for industrial level production will be more realistic in achieving the successful patent due to less number of existing protocols.

Efficacy of plant-based vaccines has been very well shown in animal models. Large number of reports exists that show many potential vaccine candidates and their high immunogenicity in different animal models (Rybicki, 2014). Now the need is to investigate immunogenicity and safety of some prominent vaccine candidates in humans. For this purpose, relevant medical groups and industries can be involved in the design of research projects so that resources and funding may become available for human clinical trials.

## Author contributions

MW, JG developed the concept and drafted the manuscript. MW and HI drew the figure and finalized the table. BM and AL contributed toward the concept and revised the manuscript critically. All authors have read and approved the final version of manuscript.

### Conflict of interest statement

The authors declare that the research was conducted in the absence of any commercial or financial relationships that could be construed as a potential conflict of interest.
